# Reformulating partially hydrogenated vegetable oils to maximise health gains in India: is it feasible and will it meet consumer demand?

**DOI:** 10.1186/1471-2458-13-1139

**Published:** 2013-12-05

**Authors:** Shauna M Downs, Vidhu Gupta, Suparna Ghosh-Jerath, Karen Lock, Anne Marie Thow, Archna Singh

**Affiliations:** 1 Menzies Centre for Health Policy, University of Sydney, Victor Coppleson Building (D02), Sydney, NSW 2006, Australia; 2 Indian Institute for Public Health (Delhi), Public Health Foundation of India, Plot No. 34, Sector - 44, Institutional Area, Gurgaon 122002, Haryana, India; 3 London School of Hygiene and Tropical Medicine and Leverhulme Centre for Integrative Research on Agriculture and Health, London, UK; 4 All India Institute of Medical Sciences, Ansari Nagar, New Delhi-29, India

**Keywords:** Trans fat, Nutrition policy, Food industry, Dietary fat, Public health nutrition

## Abstract

**Background:**

The consumption of partially hydrogenated vegetable oils (PHVOs) high in trans fat is associated with an increased risk of cardiovascular disease and other non-communicable diseases. In response to high intakes of PHVOs, the Indian government has proposed regulation to set limits on the amount of trans fat permissible in PHVOs. Global recommendations are to replace PHVOs with polyunsaturated fatty acids (PUFAs) in order to optimise health benefits; however, little is known about the practicalities of implementation in low-income settings. The aim of this study was to examine the technical and economic feasibility of reducing trans fat in PHVOs and reformulating it using healthier fats.

**Methods:**

Thirteen semi-structured interviews were conducted with manufacturers and technical experts of PHVOs in India. Data were open-coded and organised according to key themes.

**Results:**

Interviewees indicated that reformulating PHVOs was both economically and technically feasible provided that trans fat regulation takes account of the food technology challenges associated with product reformulation. However, there will be challenges in maintaining the physical properties that consumers prefer while reducing the trans fat in PHVOs. The availability of input oils was not seen to be a problem because of the low cost and high availability of imported palm oil, which was the input oil of choice for industry. Most interviewees were not concerned about the potential increase in saturated fat associated with increased use of palm oil and were not planning to use PUFAs in product reformulation. Interviewees indicated that many smaller manufacturers would not have sufficient capacity to reformulate products to reduce trans fat.

**Conclusions:**

Reformulating PHVOs to reduce trans fat in India is feasible; however, a collision course exists where the public health goal to replace PHVOs with PUFA are opposed to the goals of industry to produce a cheap alternative product that meets consumer preferences. Ensuring that product reformulation is done in a way that maximises health benefits will require shifts in knowledge and subsequent demand of products, decreased reliance on palm oil, investment in research and development and increased capacity for smaller manufacturers.

## Background

The consumption of industrially produced trans fatty acids (TFA) is associated with an increased risk of non-communicable diseases (NCDs), especially cardiovascular disease (CVD) [1-4]. As part of the Global Monitoring Framework for NCDs, the World Health Organization (WHO) has recommended TFA elimination from the diet [5] and has called for “national policies that virtually eliminate partially hydrogenated vegetable oils (PHVOs) in the food supply and replace [them] with polyunsaturated fatty acids (PUFA)” [6]. However, little is known about the practicalities of implementing these global recommendations and whether or not low and middle-income countries will have the capacity to reformulate their products in a way that maximises health gains.

In India, PHVOs are consumed principally through *vanaspati,* a vegetable ghee used as cooking oil and in fried snacks, baked goods and street vendor foods [7]. *Vanaspati* is popular because it is a cheap and stable energy source with a long shelf life that withstands repeated heating. These properties make it an attractive product for commercial baking and frying [7,8]. In response to the high levels of TFA in Indian PHVOs (*vanaspati* and bakery shortening), the Food Safety and Standards Authority of India (FSSAI), under the Ministry of Health and Family Welfare, has proposed regulation that includes setting an upper limit of 10% (by weight) TFA in PHVOs reducing to 5% over three years [9]. The proposed limits will target PHVOs rather than the foods that contain them.

At the moment, there are four regulations that may limit industry’s ability to reformulate PHVOs to meet the aforementioned TFA limits: 1) the melting point cap of 41**°**C, 2) the classification of palm stearin as an inedible oil, 3) the permitted use of only two different oils in *vanaspati* and 4) the inability to use interesterification. The melting point cap and the inability to use palm stearin in food products was based on the belief that humans could not easily digest oils with melting points higher than body temperature; however, scientific evidence refutes this assertion [10]. Moreover, interesterification and the use of more than two oils were not permitted under the Prevention of Food Adulteration Act (now the Food Safety and Standard Act). Consultations with FSSAI, scientific experts and industry were held in 2010 at the National Institute of Nutrition to discuss the proposed TFA regulation in India. The recommendations from that committee were to remove the aforementioned regulatory barriers to facilitate product reformulation.

Although previous regulation dictated that 25% of the input oil used in PHVOs be indigenous sources, in 2003 this was removed leading to a shift from domestically produced oils that were high in mono-unsaturated fatty acids (MUFA) and PUFA to palm oil [7]. Because of the high saturated fatty acid (SFA) content of palm oil it is semi-solid at room temperature thereby requiring less hydrogenation to ensure the product texture preferred by consumers [10]. As part of the FSSAI consultation process, the *vanaspati* industry commissioned a study by Frost & Sullivan showing the relationship between melting point and TFA concentration – as the hydrogenation process proceeds TFA content first increases and then decreases as the melting point is increased [10]. In this report they show that with an increase in the melting point to 47-48**°**C, the TFA content of PHVOs with palm oil as the main ingredient will decrease to 15% whereas for soybean oil the TFA content would decrease to 36% - suggesting that the higher the content of SFA in the input oil, the lower the degree of hydrogenation required and the lower the TFA levels to get the desired texture [11].

Trans fat regulation has been effective in high-income countries and many products have been reformulated without substantially increasing saturated fatty acids (SFA) [12-15] – through use of trait-enhanced oils, interesterification, fractionation and blending [16-18] – it is not known whether industry in low and middle-income countries, such as India, will be able to use the same approaches. Industry might simply shift from PHVOs to high SFA fats such as palm oil or increase the degree of hydrogenation of native oils – partial hydrogenation of oils turns the cis-unsaturated fatty acids (MUFA and PUFA) into trans-unsaturated fatty acids, but full hydrogenation turns all unsaturated fatty acids (the original cis and the intermediate trans fatty acids) into SFA. This would decrease the TFA but increase the SFA content of vanaspati. Other methods might be used to reduce TFA while limiting the increase in SFA. Trait-enhanced oils (i.e., high-oleic sunflower oil) are fats modified via selective breeding or biotechnology to increase the oxidative stability required in frying and baking [19]. Interesterification modifies the melting point and crystallisation of the fat by rearranging the fatty acids, producing TFA free fats with desirable physical properties [19]. Fractionation involves splitting fats into their solid components (i.e., palm stearin). The fractions can then either be used alone or in combination with other fats [19]. Blending interesterified oils with liquid oils high in PUFA can result in TFA free products with relatively low SFA levels [17]. Research has been conducted to determine the most appropriate technologies for specific product requirements [7,18,20].

A collision course exists in India where the public health goal to replace PHVOs with PUFA are opposed to the goals of industry to produce a cheap alternative product that meets consumer demands. The aim of this study was to examine the technical and economic feasibility of reducing TFA (to be consistent) in PHVOs and reformulating it using healthier fats.

## Methods

This study reports on qualitative interviews of Indian manufacturers of *vanaspati* and technical experts working in an NGO and government. It is part of a larger project that aims to examine awareness and use of TFA, and the feasibility of its removal from the Indian food supply from the perspectives of manufacturers, retailers and consumers. Ethics approval for the study was obtained from the University of Sydney and the Public Health Foundation of India’s Institutional Ethics Committees. All interviewees provided written informed consent.

Semi-structured interviews were conducted with respondents who had technical expertise on the use of PHVO in the Indian food industry. Industry interviewees were either the Technical or Research and Development Directors of the companies. Study participants were recruited using purposive sampling. To provide a variety of perspectives, representatives of manufacturing companies of varying sizes (including multinationals and smaller companies) were included. All potential interviewees were first contacted via telephone. Initial interviewees were identified by one of the researchers, and subsequent interviewees were recruited using chain of referral snowball sampling [21]. In addition, to identify interviewees from smaller regional companies, potential participants were contacted from a list of all the Indian *vanaspati* manufacturers; the list was obtained from the Indian Vanaspati Producers’ Association. Of the 79 manufacturers contacted, we were unable to make contact with 30 (mainly due to incorrect contact information) and 15 did not manufacture *vanaspati*. Of the remaining 34 companies, ten participated in the study; the remaining companies either refused to participate or did not return our phone calls. Many potential participants from smaller companies seemed suspicious of the intent of the study and declined to participate, resulting in a smaller than planned sample size.

Overall, ten interviews with Indian manufacturers of PHVOs were conducted. In addition, we recruited one person who was an oil technologist who presided over an oil technologist non-government organisation (NGO), and two government technocrats who work on this food policy area at the national level. Two additional manufacturers were interviewed but later decided to rescind consent from the study resulting in a total of 13 interviews. Given that the aim was to get perspectives from both smaller and larger companies, the market share of the participating players varied. However, two of the manufacturers with the highest market share participated in the study.

Interviews were conducted between May and August 2012 in Delhi, Mumbai, Jaipur, Rudapur and Rajpura, India. The majority of interviews were conducted face-to-face, although four were completed via telephone due to travel constraints around India. Two researchers (SMD and AS) conducted all of the study interviews; face-to-face interviews were usually conducted with both of the interviewers present. Telephone interviews were conducted by one of the two interviewers. The interviewers had been previously trained in qualitative methods and had experience conducting interviews. Interviews averaged 40 minutes and were conducted in English, with the exception of one that was completed in Hindi and subsequently translated to English.

The interview was based on the technical and economic feasibility of reducing TFA in PHVOs and replacing it with healthier oils (i.e., PUFAs). The interview guide topics were based on a feasibility assessment tool used to assess policy options to improve diets in the Pacific Islands [22] and was adapted for the context based on discussion with food technology experts in India. The interview guide is reported in Table 1. Five experts in the study area reviewed the interview guide prior to finalising its content; however, it had not been previously validated. Interviews were audio-recorded or detailed notes were taken, depending on whether the interviewee consented to being recorded. Interviews that were audio-recorded were transcribed verbatim. Interview transcripts and detailed notes were analysed using NVivo software (version 9; QSR International, Doncaster, Victoria, Australia). An inductive qualitative analysis was conducted by SMD. Data were first open-coded and these codes were subsequently organised based on key themes, which were later agreed upon by the research team.

**Table 1:** Interview topics based on technical and economic feasibility of reformulating partially hydrogenated vegetable oils in India

**Main topic**	**Interview questions**
Trans fat and health	What is your understanding of the health issues related to – *vanaspati*; - other cooking oils; - trans fat
Company characteristics	What are the main products that your company produces?
	Which of the products that your company produces contain hydrogenated oils?
	Where do you obtain your raw materials? (which areas, types of producers)?
Trans fat regulation	What do you know about the proposed trans fat regulation?
	How will the proposed trans fat regulation affect your company? Will it affect your profit margins?
	Could the government do anything to help (i.e., incentives, subsidies, etc.) make the transition to low trans/trans fat free products easier?
Feasibility	How feasible is product reformulation of *vanaspati* (or shortening/spreads)?
	Can you comment on the:
	Technological feasibility
	Availability of replacement oils
	Economic feasibility
	What considerations will have to be made in terms of the cultural/social acceptability of alternative products?
	What will be the main barriers or challenges associated with product reformulation?
Costs	What are the current costs associated with the production of *vanaspati*?
	Which additional costs will be involved in the reformulation of *vanaspati* (or shortenings)?
	How will these costs differ with a limit of 10% vs 5% trans fat in PHVOs?

## Results

Surprisingly all interviewees indicated that TFA was unhealthy and its consumption should be limited. However, several interviewees stated that consumption of TFA and total fat is low and that fat consumption is not as problematic in India as compared to other countries. Key themes that arose in the interviews about the feasibility of reducing TFA in India related to consumer preferences, regulatory issues, the availability of oils and both the technical and economic feasibility. Although interviewees from government, a NGO and industry were interviewed there were no major differences in their views regarding the feasibility of production reformulation.

### Consumer preferences

Interviewees suggested that consumers prefer *vanaspati* products that are hard, white and granular (bakery shortening has the “grains” removed) although there are regional variations in preference. These perceived preferences were stated as one of the reasons for the challenges in terms of product reformulation of PHVOs, as industry would need to make a product that has a hard texture, which remains semi-solid even in the hot temperatures of the summer months.

Many interviewees mentioned that it is difficult to maintain the “hardness” that consumers demand while reducing the TFA in PHVOs. Some interviewees suggested that people “need to be educated” not only about the potential negative health effects of ‘hard’ *vanaspati* products (and TFA) but also the effects of replacement with palm stearin and its effect on SFA.

The potential for SFA to increase when TFA is reduced was not seen as a widespread concern by manufacturers. One interviewee did note that increasing use of palm stearin would make “the saturated fat acid go up and that is not a healthy oil”, whereas others said palm was a “healthy oil” and an “agreeable choice” for product reformulation.

Interviewees indicated that consumers were beginning to demand healthier products. Some interviewees noted the potential to market healthier products to these health conscious consumers in order to differentiate themselves from other companies. Although some households use *vanaspati* as a cooking fat, it is predominantly sold, as several participants noted, from “business to business” to be used as an input ingredient in processed foods. It was suggested that since the main market for PHVOs is other businesses, large companies could drive demand for lower TFA products, given their “global linkages”, thereby facilitating TFA reduction.

### Regulatory issues

Overall, industry respondents were accepting of the proposed national TFA regulation (particularly a 10% (by weight) TFA limit in PHVOs), and some were even critical of the lengthy policy process. Interviewees from multinational companies indicated that the regulation would lead to a more level playing field, which could potentially benefit them. All interviewees mentioned the need to align Indian regulation with international standards (i.e., CODEX). Specifically they were concerned about the current cap on the melting point, the classification of palm stearin as inedible oil, and the inability to use enzymatic and chemical interesterification. However, interviewees from both industry and government noted that it was likely that these “archaic laws” would be removed when the government enacts the proposed TFA regulation, as suggested during consultations, given that the melting point cap results in a reliance on use of TFA in order to ensure product acceptability. Although palm stearin is currently not permitted as input oil for PHVOs, it was suggested that many companies were using it to make a very hard product that was described as being “like stone” or a “brick”.

There was a concern that there would be challenges with the implementation, monitoring and compliance of the proposed new regulation. Several interviewees stated that companies were already “violating the norms” for the melting point cap and palm stearin so compliance may remain a problem in the future: “today people violate and tomorrow they will violate”.

One interviewee noted the need for greater transparency and suggested publishing the results of food inspector monitoring of *vanaspati* quality in order to empower consumers to make informed decisions. Interviewees noted that although by law, their product should be tested once per month by food inspectors, their products are often not tested regularly. Moreover, one interviewee indicated that there had been no reported violations of the melting point regulation in the past few years even though, with the exception of a few companies, the interviewee suggested they were all exceeding the melting point cap regulation. One multinational company who had been abiding by the regulation said that they were producing a product that was not acceptable to consumers because they could not make it hard enough without violating the regulation, something that they would not do given their own company’s “ethics” leading them “to comply with every law in the land”. Table 2 provides supporting quotes regarding the aforementioned regulatory issues.

**Table 2:** **Supporting quotes related to regulatory issues for *vanaspati *** **manufacturing**

**Key theme**	**Quote**
Lengthy policy process	“We have always seen that any regulation change in India takes little longer and we have…this both famous and infamous process of far too much consultations”.
Melting point regulation	“I am forced to incorporate trans [fat] into my product…as a technical person I know that the trans [fat] is not good and the whole world is moving towards not having trans but I am forced to do that”.

### Replacement oils

There was consensus among interviewees that the production of *vanaspati* “is primarily being driven by the cost and the functional application of the product”. Some interviewees described the input oils as the costliest component of the production process, resulting in its selection being based on cost and availability. Palm oil is cheaper and more widely available than the domestically produced crops leading to industry’s increased reliance on it. In fact, there are now special ports used by larger companies to import palm oil. Some interviewees noted that the companies importing crude palm oil could then separate it into its fractions at their processing plants. Palm stearin, a hard fraction of palm oil that is the least costly, could then be used to improve the texture of PHVOs – even though the government currently does not permit its use.

One interviewee stated that the palm oil lobby pushed TFA onto the Indian agenda. Because palm oil is semi-solid it could replace PHVOs in some products. As industry moved towards use of palm oil as the main input oil in *vanaspati* and bakery shortening, interviewees noted that the total TFA in *vanaspati* decreased. When industry was using “soft oils” as the input oils into *vanaspati* they had to hydrogenate the products more to ensure that it became the desired texture (i.e., semi-solid). Given that palm oil is already semi-solid it does not require the same degree of hydrogenation as more liquid vegetable oils, interviewees indicated that it has led to changes in consumption patterns; palm oil and SFA consumption are increasing and TFA consumption from *vanaspati* is stabilising or even decreasing.

Most interviewees did not see the availability of alternative oils as being problematic, since they planned to continue to use palm oil as the input oil in their reformulated products. However, one interviewee did suggest the need to establish a supply of cheap oil for the masses that was healthier, such as canola, and another interviewee proposed the restructuring of agriculture policies to enable a shift to oils other than palm. Supporting quotes regarding the availability of oils are reported in Table 3.

**Table 3:** Supporting quotes relating to the replacement oils for product reformulation

**Key themes**	**Quotes**
Use of palm oil	“The main raw material is 90-95% only palm oil. There [is] no other option because indigenous oil prices are very high”.
Palm oil import	“The major players they have shifted their production to Kandla itself, the importing place [for palm oil], because they have got the benefit of the logistic so they can have [a] better price, manufacturing cost and all that”.

### Technological feasibility

Interviewees all agreed that the proposed TFA regulation was both technologically and economically feasible if the melting point regulation was removed; however, there will be challenges in terms of creating a product that meets consumer preferences. “I mean, this requires no rocket science and you don’t have to reinvent the wheel, the world has done it we can do it too, as simple as that”. Manufacturers indicated that they could reduce TFA in their products to 10%, given an increase in the melting point, without having to adopt new technology. This would entail increasing the hydrogenation of the product, which leads to a decrease in TFA and an increase in SFA. However, the larger companies mentioned a need to invest in new equipment and research and development to bring down the TFA to 5%, while keeping the organoleptic properties (i.e., a product that is hard) that consumers demand. One company indicated that they had previously produced a *vanaspati* product that was low in TFA but the product failed because the texture was not acceptable to the consumer.

The use of full hydrogenation, interesterification, fractionation and blending of soft oils with harder fractionations were the main ways that manufacturers planned to decrease TFA in their products. Interviewees often noted that they would use these different technological approaches in combination and based the appropriateness of the different technologies on cost and perceived consumer acceptability. More specifically, there were various pros and cons to using the different technologies attributed to the product cost, hardness, stability and shelf life, and potential health effects. The use of interesterification was identified as a way to increase the use of “soft oils” (i.e., PUFA); however, it was noted that this would still result in increased costs from both the use of the technology itself and the input oils. There was disagreement about whether interesterification would result in hard enough products – some interviewees deemed this to be problematic while others did not. In terms of fractionation and blending, there was a concern by a few interviewees that this would lead to overuse of palm stearin, which would increase SFA content of food products and negatively affect heart disease rates in the future. However, other interviewees did not see any problem with the use of palm oil or its fractions. Enzymatic interesterification was seen as being more appropriate than chemical interesterification since it was believed to be more economically feasible and used a better catalyst as compared to chemical interesterification, which one interviewee described as being potentially hazardous to human health.

### Economic feasibility

One of the key points made by all interviewees was the need to “minimise costs”. Interviewees consistently stated that reducing the TFA in their products would not significantly increase their costs and would not result in lower profit margins. However, there were also industry players, particularly the multinational companies, who indicated there would be additional capital costs if they planned to use interesterification to bring down the TFA in their products without increasing SFA content.

Several interviewees noted a lack of capacity for smaller sized manufacturers, leading to “hanky panky business” by some. This lack of capacity has led multinational companies to buy-out some of the successful smaller brands, consolidating the industry, and to the closure of some *vanaspati* plants. Given the lack of capacity of smaller manufacturers, a few interviewees postulated that they would not abide by the TFA regulation once it is implemented. However, noting this should not lead to delays in the enactment of the regulation: “Even if they [smaller companies] don’t abide by the law it will not make much difference”, since the big players have the majority of the market. Despite the lack of capacity for smaller manufacturers, most of the interviewees did not think that the government should provide additional support to them to help facilitate product reformulation, except for a few interviews that suggested that the government could provide subsidies, tax rebates and/or machinery. Table 4 provides supporting quotes regarding the economic feasibility.

**Table 4:** Supporting quotes describing the economic feasibility of product reformulation

**Key theme**	**Quotes**
Reducing costs of vanaspati	“Business is so competitive, everybody has to be on their toes to cut down the cost”.
	“People have already started using this [palm] stearin…so the unscrupulous people they are selling at a very low price…we can’t play with the health of the people so we better decide to stop the production [of vanaspati]”.
Industry capacity	“They [government] talk about trans [fat] reducing, trans [fat] reducing, trans [fat] reducing but…they are not looking at the holistic picture of what it means for the industry to cope with that”.
Compliance with regulation	“If someone is not doing it…we should not make people suffer from the health point of view - let bigger companies start [complying with the regulation]”.

## Discussion

This study suggests that Indian food manufacturers and technical experts are positive about replacing TFA in *vanaspati*. They believe that it is technically and economically feasible; nevertheless, there will be challenges in meeting consumer preferences given that industry continuously strives to meet consumer demands. Although there are currently four government regulations relating to the melting point cap, permitted use of oils and interesterification that will make reformulating PHVOs difficult, it is likely that the government will take these into account when finalising the proposed regulation in order to reduce the product reformulation challenges that industry will face. However, replacing PHVOs with PUFA, as suggested by the WHO, will remain a challenge for Indian industry due to the increased costs associated with using PUFAs. As yet there is not widespread consumer knowledge in India leading to greater demand for healthy oil products by both consumers and businesses. It is likely that reformulation of PHVOs will replace TFA with SFA.

### Replacement oils

The removal of PHVOs from the food supply is likely to lead to a shift in palm oil consumption [20]; this is promoted by a strong palm oil lobby. Although the surge in palm oil use has likely resulted in a decrease in TFA levels in PHVOs such as *vanaspati*, SFA levels may have increased concomitantly. As Indian industry moves to further reduce TFA in PHVOs, it is likely that they will continue to rely on palm oil. Although this will result in modest improvements in heart disease risk, greater effects would be observed with replacement using PUFA [1,23]. In 1987, the Mauritian government intervened to switch cooking oil from palm to soybean oil, resulting in a significant decrease in SFA and increase in PUFA, corresponding to improvements in serum total cholesterol levels [24]. Moreover, a recent study found a link between palm oil consumption in low and middle-income countries and increased risk of mortality due to heart disease [25]. Other studies, including two meta-analyses, have also found adverse effects of consuming SFA such as palmitic acid [26-28]. Nonetheless, there continues to be support for its use in product reformulation by industry.

Interestingly, the French government recently proposed setting a 300% tax on palm oil import which led to considerable opposition from the Malaysian Palm Oil Council [29]. There is an emerging trend for companies to resist using palm oil in some high-income countries due to the environmental and health impact of its consumption [30]. In fact, front-of-package logos indicating palm oil-free products are now available in Europe [30]. This growing trend may be the impetus for greater investment in alternatives to both PHVOs and palm oil; however, it is likely that palm oil will continue to be used in low and middle-income countries until consumers increase demand for products that are both low in TFA and SFA. Because there has been considerable interest in the environmental impact of palm oil production [31], highlighting the environmental as well as health concerns related to palm oil production and consumption may get more consumer buy-in.

Although there is limited information about product reformulation to reduce trans fat in low and middle-income countries, a study examining industry’s response in Latin American countries found similar challenges to those reported in this study. For example, challenges in the availability and cost of PUFA and concern about consumers sensory preference for some products [32]. Ensuring that TFA reduction does not lead to an increase in SFA will require active engagement and collaboration among the government, the agriculture sector and Indian industry. Previous research has suggested that leadership and commitment from senior management of industry, collaboration across the supply chain and adequate supply of alternative oils, increased demand for healthy products, increased media coverage and regulation are needed to enable healthier product reformulation [18]. Currently there is insufficient demand for low TFA and SFA products in India, which gives industry little incentive to reformulate in a more healthful way, especially considering the increased costs associated with use of PUFA. Nevertheless, it is important to note that energy intakes remain low for a significant portion of the Indian population and food security remains a problem for many, making the need for low priced fats essential [33].

### Technical feasibility

Although industry and technical experts indicated that it was technologically feasible to reformulate PHVOs in India to reduce TFA, they will need to invest in research and development to reduce TFA levels to 5%. In high-income countries, industry replaced PHVOs with different types of oils and fats based on the product functionality required [7,18,20]. For example, PHVOs for frying were often replaced with high MUFA or PUFA liquid oils, whereas interesterification of hard fractions with unsaturated oils has been used for solid fats such as bakery shortening [18,20,34]. We suggest adopting a similar approach in India; however, this will require educating the consumers, and perhaps industry members, about the healthiness of “soft” unsaturated oils and strengthening supply chains to increase availability and decrease costs of these oils. In the past, Indian industry was required to use a proportion of domestic oil in *vanaspati* production. We recommend that the government consider re-enacting similar regulation to incentivise the use of PUFA in product reformulation, given that most domestically produced oils are high in these fats. There is currently a limited supply of suitable alternative oils to PHVOs that are low in SFA [20]; however, if the Indian government required industry to use domestic oils, it is likely that supply chains would be strengthened.

### Economic feasibility and capacity

Multinational companies tend to have better capacity to adopt new technology as compared to smaller manufacturers. Many large companies have research and development facilities in North America or Europe, and have already begun adopting technology such as interesterification in those countries. In fact, multinational companies have developed zero TFA low SFA bakery shortenings catering to their American and European markets [35,36], suggesting that it is possible to bring similar products to India. Moreover, a food technology study conducted in Iran (where no current trans fat regulation exists) demonstrated that low TFA *vanaspati* could be produced using a combination of blending and interesterification creating a product with reasonable SFA levels and the same organoleptic properties demanded by Indian consumers [37]. However, smaller companies may not have the resources to invest in the necessary technology to create a product that is both low in TFA and SFA. Surprisingly, this study found little support for government initiatives to help smaller businesses with this process. New York City and the province of British Columbia, Canada provided support to small businesses to help enable the shift towards TFA free cooking oils and fats in response to regulation in those jurisdictions [38-40]. Moreover, in the United States, smaller companies were given extra time to comply with labelling regulation given the added difficulty they would face as compared to larger companies [39]. For Indian companies who do not have the financial means to adopt new technology, they may be forced to sell consumers a product that is less acceptable, fail to comply with the regulation, or sell or close their businesses. Interestingly, there was a trend towards multinational companies purchasing the more successful smaller PHVO businesses. One of the potentially positive aspects of this trend is that multinational companies may be less likely to stray from regulation. Therefore as more and more smaller PHVO businesses are purchased by the larger companies, this may help facilitate compliance with regulation and ease the potential enforcement strains that the country may face when the TFA regulation is enacted [8]. On the other hand, it is important to support smaller manufacturers and we suggest providing incentives to help them to better comply with regulation.

One of the main limitations of this study was the small sample size; however, our respondents included the companies with the highest market share for PHVOs in India, which strengthens the transferability of the study findings. Another limitation of this study was our use of a non-validated interview guide. Nevertheless, we ensured that the interview guide was reviewed by experts in the field prior to using it in this study.

## Conclusions

Reformulating PHVOs in India is technically and economically feasible; however, there will be challenges in terms of producing a product that meets consumer demand. Ensuring that product reformulation is done in a way that maximises health benefits will require shifts in knowledge and subsequent demand of products, decreased reliance on palm oil, investment in research and development and increased capacity for smaller manufacturers. In order to address these issues, we recommend that industry use different input oils depending on the given product’s functionality requirements. We also recommend that the government should require PHVO manufacturers to use domestic oils, as has been previously done in India, to increase uptake of PUFA use and provide support to smaller companies through use of incentives given that without support they will struggle to comply with regulation. Lastly, it will be important for India to ensure that the TFA limit in PHVOs is decreased from 10% to 5% (by weight) after three years, as proposed in the regulation, since industry will likely not adopt innovative technology (i.e., interesterification and blending) that would allow the use of PUFA with the limit of 10%, thereby decreasing the potential health impact of the proposed regulation. Continued monitoring of industry’s progress will be essential.

## Abbreviations

NCD: Non-communicable disease; PUFA: Polyunsaturated fatty acids; PHVO: Partially hydrogenated vegetable oils; TFA: Trans fatty acids; FSSAI: Food Safety and Standards Authority of India; SFA: Saturated fatty acids.

## Competing interests

The authors declare that they have no competing interests.

## Authors’ contribution

SMD participated in all aspects of this study. VG assisted with data collection and preparation of the manuscript. SGJ, KL and AMT contributed to the study conception and preparation of the manuscript. AS participated in the study conception, data collection and preparation of the manuscript. All authors read and approved the final manuscript.

## Pre-publication history

The pre-publication history for this paper can be accessed here:

http://www.biomedcentral.com/1471-2458/13/1139/prepub
